# Valorisation of Agri-Food Waste for Bioactive Compounds: Recent Trends and Future Sustainable Challenges

**DOI:** 10.3390/molecules29092055

**Published:** 2024-04-29

**Authors:** Sujeeta Yadav, Kamla Malik, Janie McClurkin Moore, Baldev Raj Kamboj, Shweta Malik, Vinod Kumar Malik, Sandeep Arya, Karmal Singh, Shikhadri Mahanta, Dalip Kumar Bishnoi

**Affiliations:** 1 Department of Microbiology, CCS Haryana Agricultural University, Hisar 125 004, India; sujeetayadav12@gmail.com; 2 Department of Biological and Agricultural Engineering (BAEN), College of Agriculture and Life Sciences (COALS), Texas A&M University, College Station, TX 77843, USA; shikhadri@tamu.edu; 3 Department of Agronomy, CCS Haryana Agricultural University, Hisar 125 004, India; 4 Department of Plant Pathology, CCS Haryana Agricultural University, Hisar 125 004, India; 5 Department of Forestry, CCS Haryana Agricultural University, Hisar 125 004, India; 6 Department of Agricultural Economics, CCS Haryana Agricultural University, Hisar 125 004, India

**Keywords:** agri-food wastes, bioactive compounds, valorisation, encapsulation, sustainability

## Abstract

Worldwide, a massive amount of agriculture and food waste is a major threat to the environment, the economy and public health. However, these wastes are important sources of phytochemicals (bioactive), such as polyphenols, carotenoids, carnitine, coenzymes, essential oils and tocopherols, which have antioxidant, antimicrobial and anticarcinogenic properties. Hence, it represents a promising opportunity for the food, agriculture, cosmetics, textiles, energy and pharmaceutical industries to develop cost effective strategies. The value of agri-food wastes has been extracted from various valuable bioactive compounds such as polyphenols, dietary fibre, proteins, lipids, vitamins, carotenoids, organic acids, essential oils and minerals, some of which are found in greater quantities in the discarded parts than in the parts accepted by the market used for different industrial sectors. The value of agri-food wastes and by-products could assure food security, maintain sustainability, efficiently reduce environmental pollution and provide an opportunity to earn additional income for industries. Furthermore, sustainable extraction methodologies like ultrasound-assisted extraction, pressurized liquid extraction, supercritical fluid extraction, microwave-assisted extraction, pulse electric field-assisted extraction, ultrasound microwave-assisted extraction and high hydrostatic pressure extraction are extensively used for the isolation, purification and recovery of various bioactive compounds from agri-food waste, according to a circular economy and sustainable approach. This review also includes some of the critical and sustainable challenges in the valorisation of agri-food wastes and explores innovative eco-friendly methods for extracting bioactive compounds from agri-food wastes, particularly for food applications. The highlights of this review are providing information on the valorisation techniques used for the extraction and recovery of different bioactive compounds from agricultural food wastes, innovative and promising approaches. Additionally, the potential use of these products presents an affordable alternative towards a circular economy and, consequently, sustainability. In this context, the encapsulation process considers the integral and sustainable use of agricultural food waste for bioactive compounds that enhance the properties and quality of functional food.

## 1. Introduction

Currently, agricultural challenges revolve around meeting the needs of global consumers for sufficient food production. However, the emphasis must be on implementing sustainable practices to prevent the generation of pollution and waste stemming from agricultural activities. Likewise, the world population has risen from 3.7 billion to 7.9 billion in the last 50 years, and according to a new United Nations report, in 2030, it is expected to reach 8.6 billion, 9.8 billion in 2050 and 11.2 billion in 2100. However, high production of crops produces huge amounts of agricultural waste. These wastes release hazardous substances into the soil and water and release greenhouse gases (GHGs) into the atmosphere; about 21 to 37% of GHG emissions are emitted by the agricultural sector [1,2]. The agri-food industry also generates a huge quantity of organic waste and biomass [3,4], which not only creates safe disposal issues but also has an adverse impact on the environment and society. To cope with these problems, there is an urgent need to extract and recover value-added products (bioactive compounds) from waste by using different technologies and utilization of these compounds to develop various valorised products, including functional foods or dietary supplements. Agri-food wastes are also an abundant source of natural and low-cost biomolecules, including carbohydrates, lipids, proteins, vitamins, etc. [5]. Therefore, the cost-efficient utilization of agri-food waste and byproducts will contribute to reducing environmental concerns and playing a prominent role in food processing, agriculture, cosmetics, pharmaceutical industrial applications and succeeding sustainability and circular economy concepts with innovative approaches [6].

**Figure 1:**
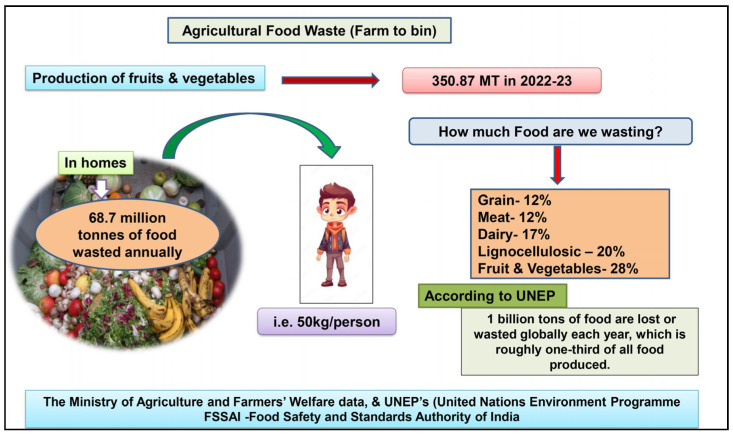
Pictorial representation of production of agricultural food waste in world.

According to the UNEP (United Nations Environment Programme)’s food waste index report, 68.7 million metric tonnes of food are wasted annually in Indian homes, or about 50 per person. Per the National Resources Defence Council (NDRC) report, in Asia, about 1.34 billion metric tons of foods are wasted. According to the Food Safety and Standards Authority of India (FSSAI), about one third of all types of food produced are wasted or spoilt before being eaten (Figure 1); almost 40% of fresh foods are spoilt during transport and storage.

Thus, there is a dire need to valorise agricultural wastes into different valuable products with the ‘zero waste concept’. The United Nations’ 17 Sustainable Development Goals (SDGs) were created to spur global economic growth and help countries tackle these issues that affect society and the environment. The United Nations has set forth a global agenda aiming for sustainable development by 2030. One of its key objectives, labelled Objective 12—responsible consumption and production, emphasizes the need for collaborative action among nations to diminish both food loss and food waste. Food loss encompasses quantifiable losses occurring from production through to the retail stage, whereas food waste pertains to levels within retail and consumption. Therefore, this review highlights the value of agri-food wastes for the extraction and recovery of bioactive compounds and their potential applications in the food sector to avoid post-harvest loss.

## 2. Agri-Food Waste as a Source of Natural Bioactive Compounds (BCs)

**Figure 2:**
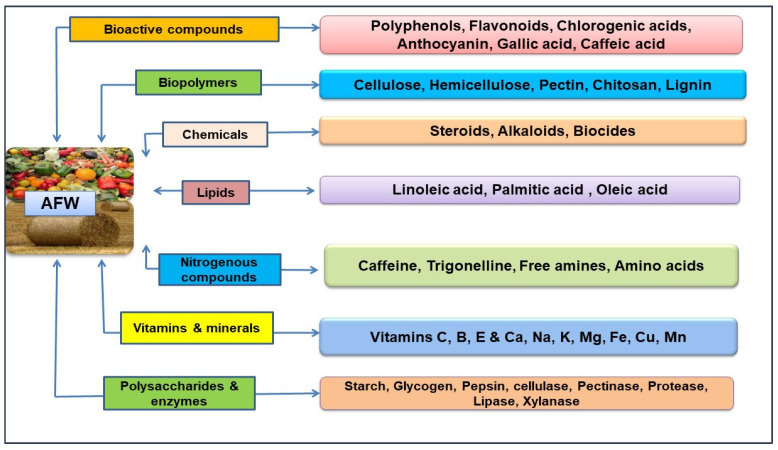
Different type of compounds present in agri-food wastes (AFW).

Agricultural food residues (plants, fruits, vegetables, cereals, etc.) produce billions of tonnes of waste at various stages of the food chain (production, handling, storage, processing, packaging, distribution, marketing and consumption) and have a negative impact on the environment and human health. In this way, there is a need to develop an innovative methodology for the utilization of these wastes into high-value-added products. Moreover, agriculturally derived lignocellulosic biomass like straw, stalk, stover, husk, bagasse, leaves, seeds, etc. [7,8] consists of cellulose (40–60%), hemicellulose (10–40%) and lignin (15–30%). Generally, agri-food residues including peels, pulp, seeds, shells, pomace, husks, straws, barks, bran and leaves [9] and the inedible parts of fruits (peel, pulp, seeds, pomace, etc.) and vegetables (stems, leaves, husks, shells and seeds) are organic materials which constitute an important source of phytonutrients (bioactives) [10,11]. A bioactive compound is a natural biological phytoconstituent or substance that can activate a physiological response in a living organism and have many beneficial effects on human health as well. Agri-food waste having different type of compounds like phenolics, peptides, polyphenols, tannins, alkaloids, carotenoids, terpenes, flavonoids, flavanols, anthocyanins, essential oils, etc., along with carbohydrates, enzymes, fatty acids, dietary fibres, lipids, biopolymers, nitrogenous compounds, minerals, vitamins, amino acids, chemicals and other phytoconstituents (Figure 2) [12,13].

Natural bioactive compounds encompass a diverse array of structures and activities, holding significant potential for the advancement of nutraceuticals, functional foods and food additives. While certain molecules, like polyphenols, are abundantly present in nature, others occur in such minute quantities that they require large-scale harvesting and chemical synthesis, which can be economically unfeasible. Phenolic compounds have different categories, like flavonoids, phenolic acids, tannins, quinones, glycosides, etc. Among these categories, flavonoids have different sub-classes: flavonols, flavanones, isoflavones and anthocyanidins, which constitute the predominant group of plant phenolics. Another category of bioactive compounds is phenolic acids, including hydroxybenzoic and hydroxycinnamic acids [14]. These components can be obtained through separation, identification and characterization processes for potential utilization across various industries such as food, pharmaceuticals, cosmetics and textiles [15,16]. These bioactive compounds are associated with a reduced risk of cancer, cataracts, Alzheimer’s, Parkinson’s disease, aging-related disorders and heart-related diseases. Their notable antioxidant and antimicrobial properties contribute to defending against chronic ailments, inhibiting the formation of cancer-causing agents and maintaining the immune system. These compounds can also prevent food spoilage and the proliferation of harmful microorganisms (antimicrobial activity) and, in some cases, act as a dietary supplement or provide health benefits. They offer benefits as additives in functional foods or as dietary supplements. Additionally, natural antioxidants and colour compounds hold promise as superior alternatives to synthetic antioxidants, applicable across pharmaceutical and processing industries [17,18]. Presently, the majority of research endeavours are concentrated on crafting novel value-added products. The prominence of value-added technologies highlights the extensive utilization of agricultural waste, particularly within the realm of food pharmacy. The extraction technologies are categorized according to their extraction efficiency, cost-effectiveness and sustainability. Several extraction processes are followed for the recovery of bioactive compounds from fruit industry by-products.

## 3. Valorisation Methods for Agri-Food Wastes

**Figure 3:**
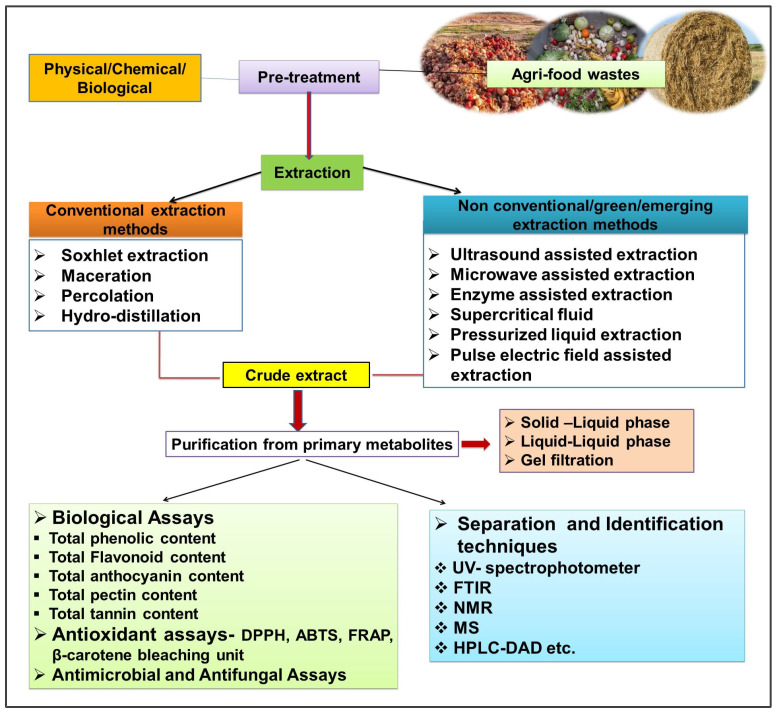
Flow chart for the extraction and characterization of bioactive compounds extracted from AFW.

The valorisation of agri-food waste is a useful and innovative technology for the production of value-added products such as bioactives, biopolymers, chemicals, nutraceuticals, antioxidants, biopeptides, antibiotics, industrially important enzymes, bio-nanocomposites, polysaccharides, lipids, vitamins, minerals, pigments, polyphenols, fatty acids, etc. that could be recovered from these wastes and used as a potential substrate for applications in food, agriculture, health, pharmaceutical, cosmetic and environment sector [11]. In addition, conventional (Soxhlet, maceration and hydrodistillation) and emerging (supercritical fluid, subcritical fluid, microwave-assisted, ultrasonic-assisted, enzyme-assisted and pulsed electric field-assisted) techniques, along with separation, isolation and identification by different analytical methods and quantification using various chromatographic and spectrophotometric methods and then identification and characterization of compounds as summarized in the flow chart shown in Figure 3.

Extraction techniques are among the most important processes for characterizing and recovering the bio-accessibility and availability of bioactive compounds from AFW. Furthermore, these are extracted into high-value products and used in food additives, nutraceuticals, therapeutics and cosmetics. However, the economic feasibility of the extraction of the high-value components must be considered. From this point of view, various nonconventional/novel techniques are used to transform or valorise waste into a value-added product.

### Conventional and Non-Conventional Extraction Methods for the Recovery of Bioactive Compounds from Agri-Food Wastes/By-Products

**Figure 4:**
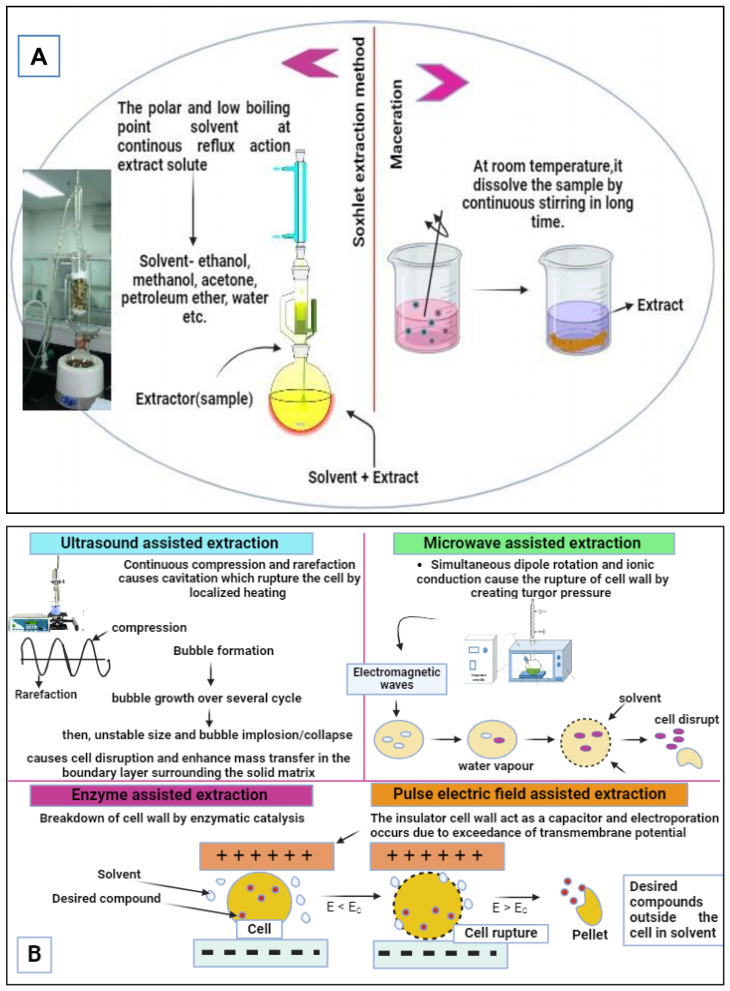
Schematic diagram detailing available conventional (**A**) and nonconventional (**B**) extraction technologies along with their respective mechanisms of action.

Beneficial compounds, particularly bioactives, are found in the innermost cellular portions of fruit by-products, and their release necessitates more targeted processes. Therefore, non-conventional extraction techniques, such as supercritical fluid extraction (SFE), pulsed electric fields (PEF), ultrasound-assisted extraction (UAE), microwave-assisted extraction (MAE), enzyme-assisted extraction (EAE) and pressurized liquid extraction (PLE), which are economically viable, safe and innovative processes are more preferable in the recent era. Figure 4 presents a schematic diagram detailing the availability of conventional and non-conventional extraction technologies along with their respective mechanisms of action. The suitability of extraction technology depends on some specific factors such as the desired purity level of the extract, the physical and chemical properties of the compound, its location within the by-product (whether free or bound within the cell), cost-effectiveness and the value of the resulting product. Prior to the extraction process, several operations or pretreatment are typically undertaken to enhance yield. Examples of these operations include washing, cutting, size reduction and drying [19]. Conventional or traditional methods encompass Soxhlet extraction, maceration, infusion, percolation, decoction, steam and hydrodistillation. Drawbacks of conventional techniques include higher organic solvent consumption, greater purity demands, increased cost, reduced extraction efficiency, extended processing durations, elevated temperatures and the release of harmful residues [20].

Various valorisation methods based on the characteristics of the waste, which are more sustainable and profitable to manage agri-food waste, arise as alternative options to obtain value-added products (bioactive).The nature and properties of the bioactive compounds depend on the techniques and extraction conditions; i.e., one technique or specific combination of techniques like ultrasound microwave-assisted extraction (UMAE) can be chosen for extraction of specific type of bioactive compounds. Aromatic compounds are basically extracted from plants using conventional methods like hydrodistillation and maceration. These techniques prove valuable in extracting challenging or low-abundance compounds, despite their extended operational durations [21]. Additionally, it is imperative to assess the economic viability of the process and its applicability, especially in sectors like agri-food and pharmaceuticals, where ensuring the production of specific compounds with low or non-toxicity is critical also. Regarding security concerns, the toxicity and presence of different solvents in the extracts, along with low yield, have led to a greater interest in green and modern extraction technologies to maximize the yield of products while minimizing any negative effects of solvents [22]. Regarding economic concerns, the desired phytochemicals should be extracted using appropriate methods, which include obtaining all of the value-added components for complete utilization of the agri-food waste. Table 1 describes the application of non-conventional green extraction techniques for bioactive recovery from various substrates under optimized conditions.

**Figure 5:**
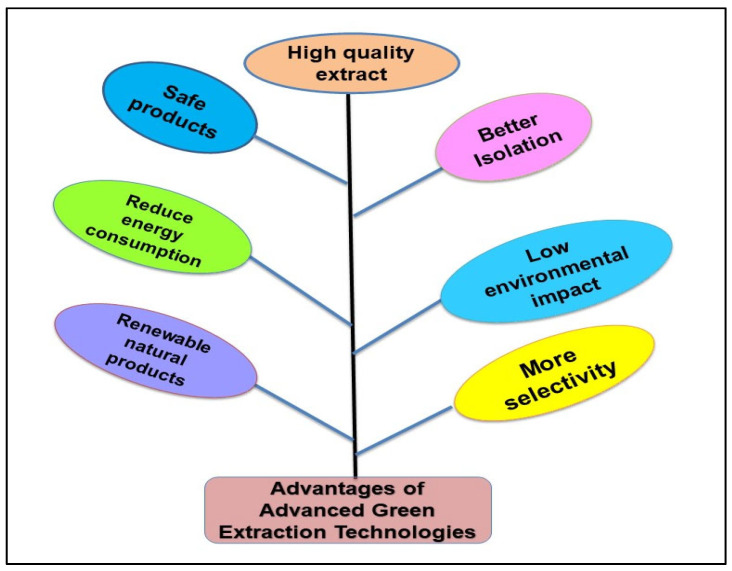
The advantages of non- conventional techniques for extraction.

Emerging extraction techniques are rooted in non-thermal principles, aiming to facilitate extraction without the risk of overheating the waste matrix, all while reducing energy consumption [36]. The innate moisture content present in agriwaste causes molecular perturbations as external energy sources. To overcome this challenge, microwave and ultrasonication methods are harnessed for residues with elevated moisture levels. When water usage proves unfeasible, ethanol emerges as the preferred solvent. The yields of bioactive compounds after extraction depend upon various factors, i.e., extraction method, physio-chemical attributes of the waste material, type of solvent, temperature, time, pressure, etc. The non-conventional extraction methods have numerous advantages over conventional methods, as depicted in Figure 5. These advanced green extraction technologies have many applications in food as well as environment.

After the completion of the extraction, the crude extracts contain different types of bioactive compounds, which are subsequently identified and quantified to further profile the specific extracted bioactive compounds. Generally, these analyses are simple colorimetric methods which determine total polyphenolics, flavonoids, betalains, glycosides, tannins, etc. [37].

Further, the potential of bioactive compounds can be evaluated on the basis of their antioxidant (radical scavenging capacity) and antimicrobial (microbial inhibition) property. The radical scavenging ability of compounds is assessed using the 1,1-diphenyl-1-picrylhydrazyl (DPPH) and the 2,2′-azino-bis (3-ethylbenzothiazoline-6-sulfonic acid) diammonium salt (ABTS) assay. Simultaneously, the ferric reduced antioxidant power (FRAP) assay gauges the bioactive compounds’ capacity to reduce the ferric ion (Fe^3+^) complex to the ferrous ions (Fe^2+^). Additionally, the oxygen radical absorbance capacity (ORAC) assay also evaluates the ability of bioactive compounds into scavenge peroxyl radicals that result in an inhibition of the decreased the level of fluorescence in the reaction [38]. In addition, different techniques are applied for separation and identification of bioactive compounds, including gas chromatography-mass spectrometry (GC-MS), ultra-high-performance liquid chromatography (UHPLC) and attenuated total reflectance infrared spectroscopy (ATR-FTIR) [39,40].

## 4. Encapsulation of Bioactive Compounds in Food and Agriculture Sectors

Agri-food wastes are organic and bio-degradable materials and a vital source of natural bioactive compounds, i.e., phytochemicals, such as phenolics, carotenoids, carotenoids, vitamins and tocopherols, that can be recovered by valorisation technologies into value-added products that have potential applications in the food, therapeutics and environmental sectors with several health benefits, low toxicity and no adverse effects [41,42]. These natural bioactive compounds are safe but sometimes have an unpleasant taste or odour, instability, low solubility, rapid release, inadequate bioavailability, vulnerability to degradation and high sensitivity to diverse environmental factors like pH, temperature, oxygen, light and moisture when incorporated into biological formulations. These factors collectively contribute to the diminishment of the nutritional and functional attributes of these compounds during storage. Therefore, to safeguard the integrity of bioactive compounds and enhance their suitability for applications in food and agriculture, encapsulation has emerged as a viable solution. Mostly of these bioactive compounds could be susceptible to an oxidative degradation, which causes the generation of free radicals that compromises their bioactivity [43].

**Figure 6:**
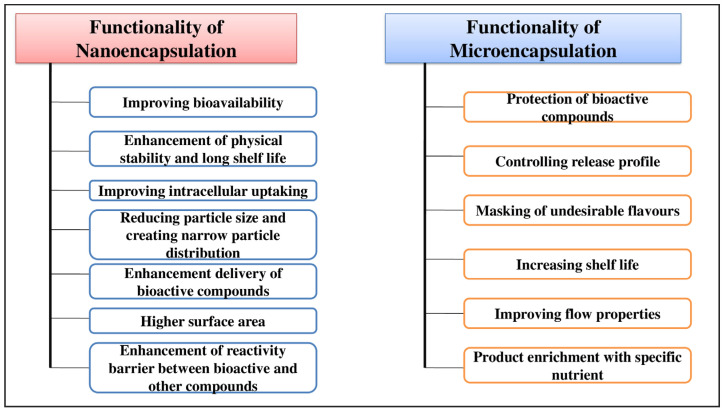
Different functionalities of nano- and microencapsulation in food and agriculture.

Encapsulation technology encompasses two primary methods: micro-encapsulation and nano-encapsulation. Both techniques offer distinct advantages for enhancing product functionality as shown in Figure 6. Nanoencapsulation and microencapsulation refer to the particle size of capsules, with nanoencapsulation involving sizes ranging from 10 to 1000 nm and microencapsulation having a size range of 3 to 800 μm [44]. Nanocapsules offer a larger surface area, which enhances solubility compared to microcapsules. Additionally, nanocapsules play a crucial role in improving bioavailability and achieving sustained release of drugs, enabling precise targeting of active compounds. These techniques involve coating with bioactive core compounds as a wall material, creating capsules that act as effective barriers against environmental and chemical interactions. They enhance encapsulation efficiency, offer substantial loading capacity, improve bioavailability, ensure stability, enable controlled sustained release and provide flavour-masking capability. These distinctive attributes collectively position nano-encapsulation as a powerful and promising venue for encapsulating bioactive compounds. The multifaceted benefits it offers contribute to its growing prominence as a preferred method for optimizing the delivery and effectiveness of bioactive compounds in various applications [45].

**Figure 7:**
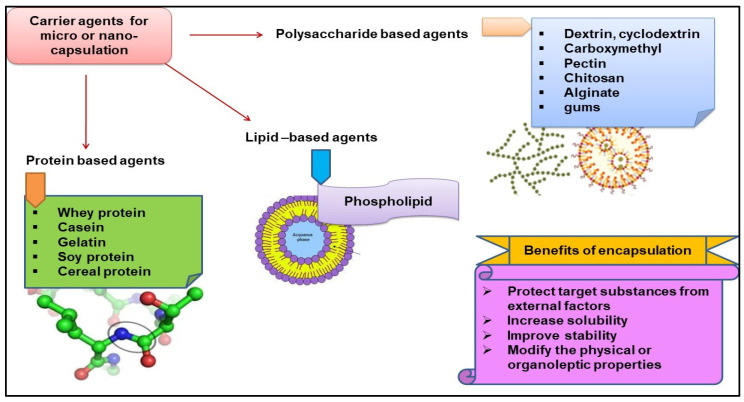
Depiction of encapsulating materials (carrier agents) used for capsulation systems.

For encapsulation, there are two pivotal aspects that play a crucial role: firstly, the careful selection of encapsulating materials, often referred to as carrier agents, to create a well-suited encapsulation system (Figure 7); and secondly, the choice of an appropriate encapsulation technique (Table 2). Many polymers are used as wall materials to protect the core, generally formed from bioactive compounds. Chitosan, gums (gum Arabic, Xanthan gum, gum acacia and shellac, for instance), maltodextrin, pectin, starch, whey protein, sodium alginate, cellulose and carboxymethylcellulose, zein, pullulan, galactomannan and sodium caseinate, among others, are used for this purpose [46]. The polymers are incorporated into food matrices with active compounds and can be integrated uninterrupted into the food matrix, having intrinsic attributes like appearance, texture and flavour. They also protect the compound from degradation: encapsulation provides a shield against chemical, physical, or biological degradation, act as safeguard of the active compounds. In terms of taste masking, a bitter or astringent taste can be effectively masked through encapsulation. Regarding enhanced stability of active compounds during both transportation and storage, handling of active compounds becomes more convenient and helps to prolonged release of bioactive compounds for long times with a specific conditions such as at different ranges of pH, temperature, etc. From the perspective of health and safety, the encapsulating agents must fulfil specific criteria. They should be officially recognized as ‘generally recognized as safe’ (GRAS) substances for use in food applications and they should demonstrate biodegradability, as stipulated by governmental organizations.

Second, the design of nano-emulsions encompasses a range of approaches, with the selection influenced by factors such as the intended final product, overall energy demands and storage prerequisites. Two primary strategies stand out for the creation of nano-emulsions are low-energy and high-energy methods [47,48]. Within these categories, various techniques can be employed to encapsulate different natural antioxidants, as shown in Table 2.

**Table 2:** Different type of techniques for encapsulation of natural antioxidants with specific nanocarriers.

Encapsulation Techniques	Methods/Techniques	Nanocarriers	Applications	Reference
Physical	Spray-drying Spray-chilling Spray-coating Supercritical micro-encapsulation Micronization Ionic gelation Freeze-drying Fluidized bed coating Centrifugal extrusion	Nano-capsules powder	Phenolic acid, carotenoids Pigments Pigments Carotenoids Nutraceutical Carotenoids, Pigments Carotenoids Food ingredients	[49]
Chemical	Interfacial polymerization Molecular inclusion Insitu polymerization	---	Nutraceutical	[50,51]
Physical-chemical	Coacervation Complex coacervation Emulsion solvent evaporation Solidification emulsion Liposomes	Hydrogel β-ciclodextrine Liposomes	Volatile flavour oils Lycopene Food ingredients Nutraceuticals	[50,51]

- Phase Separation: Utilizing controlled phase separation to encapsulate natural antioxidants within distinct phases of a system.
- Spray-Drying: Converting liquid formulations into powdered forms by atomization and drying, thus encapsulating antioxidants.
- Freeze-Drying: Preservation of antioxidants by freezing the formulation and then sublimating the frozen solvent under vacuum.
- Nano-emulsions: Formulating emulsions on the nano scale, offering efficient encapsulation of antioxidants.
- Liposomal Entrapment: Enveloping antioxidants within lipid bilayers, forming liposomes that enhance stability and controlled release.
- Coacervation: Phase separation of polymers, leading to the encapsulation of antioxidants incoacervate droplets.
- Inclusion Complexation: Formation of inclusion complexes, often with cyclodextrins, to encapsulate antioxidants.
- Ionic Gelation: Creation of gel-like structures through ionic interactions to encapsulate antioxidants.
- Solvent Evaporation: Dissolving antioxidants in a solvent, which is subsequently evaporated to leave behind encapsulated particles.
- Supercritical Fluid Precipitation: Employing supercritical fluids to precipitate antioxidants and form encapsulated particles.

Two distinct approaches, known as the bottom-up and top-down approaches, are utilized for the production of nano- and microcapsules. In the bottom-up approach, capsules are created through the self-building and self-organization of molecules, starting from small particles (nanometres) and progressing to larger encapsulated aggregates (millimetres). On the other hand, the top-down approach involves the disintegration of bulk solids, liquids, or large particles (millimetres) into smaller particles (ranging from 1 micrometre down to, for instance, 200 to 450 nanometres in the case of gelatin hydrogel particles) by using mechanical stress such as milling, shredding and grinding. This process utilizes forces like shearing, impact and compression to break down the particles. Top-down methodologies include techniques like emulsification, solvent evaporation and extrusion.

Research on the utilization of encapsulated bioactive compounds like lycopene, hydroxytyrosol and resveratrol extracted from agro-industrial waste and its by-products can be further reinforced. This research is crucial due to its significant potential in leveraging the antioxidant and/or antimicrobial properties of these compounds for various food applications, thereby contributing to advancements in the food industry. Incorporating bioactive compounds into food products poses a challenge that requires thorough evaluation, encompassing not only their nutritional value but also ensuring the chemical safety of the ingredients.

Several factors need to be considered during the design of encapsulation processes, including functionality, encapsulate concentration, target release and requirements for stability. The quantification of encapsulated compounds can be achieved through various techniques, such as high-performance liquid chromatography (HPLC), UV-vis spectroscopy and UV-vis spectroscopy. An ideal nanoparticle is characterized by maximum compound loading capacity with minimal wall material quantity. Loading of these bioactive compounds could be accomplished through two methods: incorporation and absorption. The entrapment capacity primarily relies on factors such as the solubility of the compound in the wall material, the interaction between molecules of the bioactive compound and polymer, molecular weight and the availability of functional groups [52].

Encapsulation serves various purposes, such as masking undesirable flavours, forming solid particles, reducing evaporation or volatility loss, enhancing the reactivity barrier for bioactive compounds and improving the physical stability, biological activity and shelf-life of these compounds. To extend the shelf-life of packaged foods, natural and synthetic additives with antioxidant or antimicrobial properties can be incorporated into the biodegradable material matrix.

Recent advancements in the field of nanoencapsulation and the delivery of natural bioactive or pharmaceuticals have been extensively explored. A review by [53] explored the utilization of chitosan scaffolds for nanoencapsulation and delivery. The study aimed to elucidate the mechanisms behind controlled in vitro and in vivo release in diverse biological and physiological contexts. Additionally, it highlighted various modification techniques applied to chitosan-based nanocarriers, emphasizing their potential in biomedical applications and medical uses. Well-designed micro- and nanocapsules have demonstrated that the excellent release of bioactive compounds, under thermal treatments at specific sites e.g., as in vitro gastrointestinal conditions [54] and in bioassays simulating the control of nematodes in the field [55].

## 5. Potential Applications of Encapsulated Bioactive Compounds in Agriculture and Food Industry

**Figure 8:**
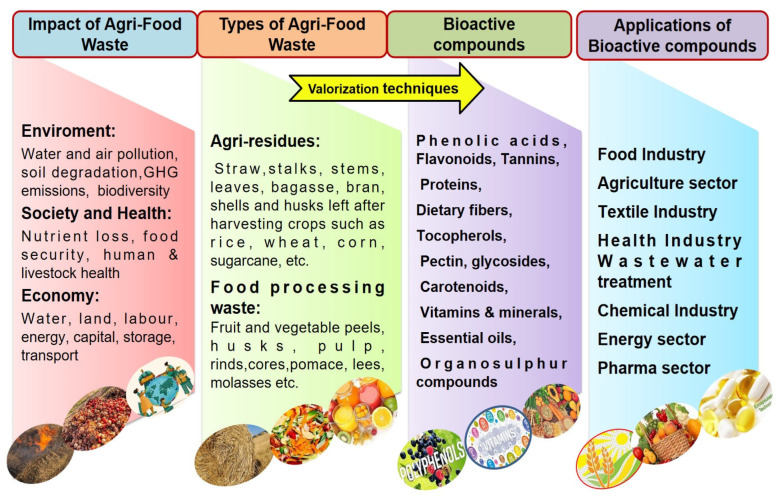
Overview of impact, types and application of bioactive compounds extracted from agri-food wastes.

**Figure 9:**
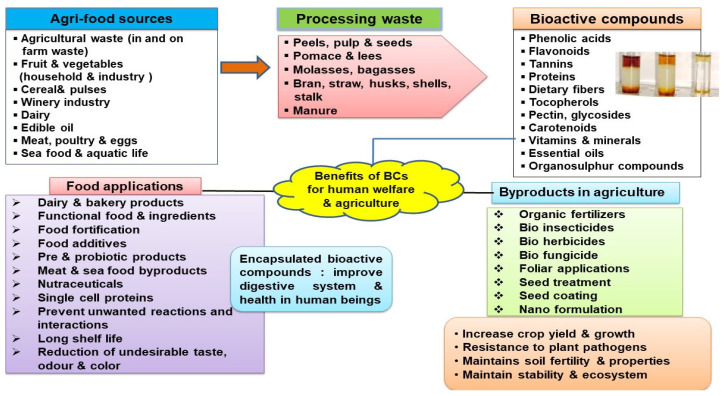
Application of encapsulated bioactive compounds in the food industry and agricultural sector.

Micro- and nano-encapsulation of bioactive compounds have led to significant advancements in various industries including food, nutraceuticals, pharmaceuticals and agriculture (Figure 8). These technologies play a crucial role in protecting a wide range of compounds such as vitamins, minerals, flavonoids, essential fatty acids, polyphenols, flavours, antimicrobials, colourants and antioxidants. Additionally, they enable precise control over the release of these bioactive compounds, ensuring their targeted delivery to specific cells, tissues or organs in the human body, thereby enhancing absorption and penetration through the gastrointestinal (GI) tract. Encapsulation plays a vital role in maintaining the biological integrity of products and protecting active ingredients from environmental factors during storage, ensuring their viability over extended periods [56]. For instance, microbial agents are often vulnerable to abiotic and biotic factors such as ultraviolet radiation and adverse temperatures, leading to a decline in their effectiveness and viability, which encapsulation helps to mitigate [57]. In agriculture, encapsulation offers numerous advantages, including reducing losses due to volatility, enhancing biological integrity, increasing efficiency, improving commercial viability and enhancing formulation stability (Figure 9) [58]. Consequently, utilization of bioactive compounds as agents for the production of bioinsecticides, biofungicides, bioherbicides and biofertilizers represents a promising approach.

In the food industry, nanotechnology has been employed in diverse ways to enhance food properties such as texture, flavour, taste, nutrients, bioavailability; colouring agents and shelf-life through the use of nanostructures (Table 3). Recently, the utilization of micro- and nanocapsules in food production plays a prominent role in a different industry such as cereal, bakery, dairy and beverages, as well as in packaging and coating applications. Additionally, these are also used as a preservative that inhibit food contamination and oxidation, further enhancing their versatile applications [59]. For food encapsulation techniques, these compounds are used as dyes (colouring), pigments, flavouring agents (candies), vitamins (vitamin A, D, E), antioxidants (citrus, grapefruit) and cereals (rice), enzymes (lipase, protease, invertase), bioactive peptide (dairy products-milk) and polyunsaturated fatty acids (omega 3, omega 6) and mineral elements (iron, calcium, potassium and others as well).

In the study conducted by [60], the impact of an active biodegradable film, fortified with encapsulated grape skin anthocyanins, on the quality of extra-virgin olive oil was investigated under accelerated storage conditions involving heat and light. When compared to a commercial polypropylene, olive oil packaged in enriched film pouches exhibited commendable oxidation stability even under accelerated thermal and photo-oxidative conditions. According to the limits established by the Codex Alimentarius, the quality of olive oil was sustained for over eight days when encapsulated, whereas oil packed in polypropylene pouches deteriorated before the fourth day of storage.

**Table 3:** The applications of encapsulated bioactive compounds extracted from agri-food wastes and used in the food industry.

AFW/By-Products	Carrier Agents	Encapsulation Methods	Encapsulated Bioactive Compounds	Model Food	Application	Reference
Carrot waste	Sodium alginate	Electrostatic extrusion	Carotenoids	Yoghurt		[61]
Pomegranate peels	Maltodextrin	Spray-drying	Punicalagin (phenolic compound)	Cookies, ice cream		[62]
Tomato peel	Whey protein	Freeze-drying	Lycopene	Salad dressing	Good functional and mechanical characteristics; reduced weight loss, increased firmness and good looks	[63]
*Byrsonima crassifolia* leaf extract	Chitosan	Coacervation	Ascorbic acid	Biodegradable film	Antimicrobial activity	[64]
Cocoa hulls	Maltodextrin	Spray-drying	Polyphenol	Biscuit		[65]
Grape skin	Maltodextrin	Freeze-drying	Carvacrol	Biodegradable film (To reduce post-harvest Loss)	Decreased respiratory rate and increased mechanical resistance	[66]
Apple pomace	Chitosan	Coacervation	Polyphenol, flavonoids, hydroxycinnamic acid, dihydroxy alkaloids	Gluten-free crackers, ice cream	Reduction of moisture loss, slowing down of respiration	[67]
Banana peel	Maltodextrin	spray-drying	Ascorbic acid	-		[68]
Grape pomace	Chitosan	Freeze-drying	Polyphenols Polyphenols	Yoghurt cheese Bread		[69]
Tamarind seed	Maltodextrin	Spray-drying	β-cartoene	Cookies and mango juice		[70]
Beetroot pomace	Maltodextrin	Emulsion	Betalain, β-cyanins	Candy, biscuits		[71]
*Curcum longa* L. root	Chitosan	Spray-drying Emulsion and ultrasonication	Curcumin	Colourant (fortified rice) Food colourant	Antioxidant activity	[72]
Tomato peel/pomace	Inulin	Spray-drying	Lycopene	-	Antioxidant activity	[73]
Black Beet root pomace	Maltodextrin	Emulsion	Cyanidin, delphinidin, malvidin, pelargonidin, peonidin, petunidin	--	Antioxidant activity	[74]
Blueberry pomace	Whey protein	Emulsion	Phenolics (p-hydroxybenzoic acid, epicatechin gallic acid), anthocyanins	-	Antioxidant activity	[75]

The increased need for biological products in agriculture these days has prompted research into new formulation methods, especially the creation of biological capsules. These bioproducts’ stability and the more reactive active ingredients, which reduce volatility-related losses, are what drives this trend [76]. Creating biocapsules with high-value compounds derived from agro-industrial by-products is beneficial commercially from a circular economy standpoint, as well as ecologically sustainable [77]. In order to counteract microbial attacks on crops during the pre- and post-harvest stages, bioactive chemicals have gained prominence as a possible organic alternative [78]. During the post-harvest stages, microbial attacks (fungi) become a challenge and cause losses of approximately 40% of the total production, requiring the application of chemical treatments to combat. Hence, the use of bioactive compounds has proved to be an excellent tool for controlling post-harvest pathogens (fungi). Due to their properties, these compounds do not induce toxicity and leave no residual effects. On the other hand, due to their antibacterial and insecticidal qualities, among others, compounds produced by microorganisms, herbal extracts and essential oils are also targeted and encapsulated [79,80]. Biotechnology has a challenge in controlling the high volatility of oils and extracts. Since it is preferred for these chemicals to release gradually and continuously regarding the target, encapsulating essential oils and volatile extracts during the development of bioproducts for agricultural applications is of significant interest [81].

Encapsulation is a novel and efficient process that involves covering one or more active substances that are coated with materials, core or internal phase and loaded in a homogenous or heterogeneous matrix which can be a shell, wall or carrier material at 1–5000 μm or nano (<1 μm) scale. After that, the molecules are entrapped and protected from the deteriorating exterior conditions like degradation, evaporation and oxidation. It is a method that improves the transport of bioactive chemicals and living cells into foods by encasing active substances within a carrier material (lipids, proteins, carbohydrates or waxes). The encapsulated materials depend on a number of variables, including their chemical and physical characteristics, suitability for the intended culinary application, impact on the final food product’s sensory and visual aspects and carrier material cost. Even though there are many processing choices, choosing an appropriate carrier system is frequently the first step in the product development process. Carriers differ greatly in terms of both price and encapsulation performance [82].

Encapsulated materials consist of a variety of polymers (synthetic or natural) [55]. In agriculture, mostly non-hydrolysed biopolymers have been used due cost effective and easily available. For examples, the hydrolysed starches used as agents in the encapsulation of Bt pesticides (*Bacillus thuringiensis*) because they are environment friendly and improved the formulated products [83]. Encapsulation has a much lower impact on the environment as well as ecosystem. Taking into account agricultural, one study cited as [84], encapsulation of *Trichoderma harzianum* spores led to improved control of phytopathogens such as *Sclerotinia sclerotiorum*, a common cause of white mould disease. By encapsulating the spores, their sensitivity to biotic and abiotic factors is mitigated, allowing for better performance in combating plant diseases. Furthermore, another study demonstrated the encapsulation of *Trichoderma* species spores in biologically based lignin for treating diseases of the vine trunk. Through in vitro tests, it was observed that the encapsulated spores remained dormant until triggered by the fungus itself at the appropriate time for germination. This controlled release mechanism ensures optimal timing for spore activation, enhancing the efficacy of disease management. Overall, encapsulation technology offers a promising strategy to improve the performance of *Trichoderma harzianum* used as a biological control agent in agriculture, particularly in the management of plant diseases such as white mould and vine trunk diseases. Similarly, one another report shown that inoculating with two strains of *Pseudomonas fluorescens* used as an alginate–gelatine capsule in potatoes used against protection from harmful soil conditions in the rhizosphere [85]. *Pseudomonas* fluorescens was encapsulated in sodium alginate with salicylic acid and zinc oxide (ZnO) nanoparticles, shown the maximum efficiency as antifungal activity against *Sclerotium rolfsii* [86]. When the biofertilizers were encapsulated with the formulations of *Burkholderia cepacia* and *Pseudomonas fluorescens* and phosphate alginate used for growth of wheat crop in semi-arid and salt-stressed areas and observed that the encapsulated crops showed better growth as compared to control [87,88]. Similarly, the biofertilizers (*Pseudomonas fluorescens* and *Azosprillumbrasilense)* encapsulated with the formulation of polymers of montmorillonite (clay mineral) and sodium alginate resulted in higher growth in crop as compared to control and slow release of the active compounds occurred. From this study observed that encapsulated techniques were contributed positive effect on the growth as well as an increased in the biomass and aerial part of the wheat plants [47,89]. Consequently, encapsulation plays a significant role in preserving and enhancing the functionality of bioactive compounds in the agriculture and food industry [90].

Here, we contrast how the cost–use ratio is affected by high- and low-cost carrier systems. At low loading levels, the low-cost carrier has an advantage. Because it can hold onto more of the active compound, the higher-cost carrier starts to look competitive at about 12% initial loading. If our goal is to minimise the delivered active cost, we will use the higher-cost carrier with a load greater than 20%. Keep in mind that the cost of the application’s active component is about doubled, even in the best of circumstances. Product developers or encapsulation experts can benefit from evaluating encapsulation from a quantitative economic perspective in a variety of ways with the initial benefits being rapid assessment of the feasibility of cost targets prior to the beginning of actual project work, to compare different encapsulation techniques that can ultimately meet cost targets are the focus of attention and to compare the cost effect of different carrier systems used with a single encapsulation technique or specific type of encapsulation on active materials of varying costs and the final application’s perceived value by the user. From the customer point of view, a substantial amount of market research is necessary as it is frequently entwined with cost [91].

## 6. Recent Trends and Challenges in Encapsulation Process

At present, consumer interest in food products with bioactive ingredients is primarily directed towards those that are free from artificial and synthetic additives. Natural bioactive compounds characterize a special group of health-related molecules that could be applied in a great variety of food classes: functional foods, nutraceuticals, dietary supplements and used as food additives, providing consumers with a natural and sustainable alternative to synthetic ones [78,92]. One of the current efforts towards increased ingredient stability, controlled release of the bioactive compounds and increased product shelf-life is the use of encapsulation technology. The main drives of the encapsulation approach are to improve the stability of sensory properties, solubility, bio-availability, retention of bioactive properties and microstructure, reduction of hygroscopicity and increase the shelf-life of desired products. Edible films or coatings are mainly made up of carbohydrates (cellulose, modified starch, alginate, agar, maltodextrin, cyclodextrins, pectins and chitosan), proteins (casein, corn zein, whey protein, gelatine, wheat gluten and soy protein) and lipids (waxes, paraffins, hydrogenated vegetable oils, phospholipids, acetoglyserides, shellac resins, mono- and triglycerides) through common encapsulation approaches such as spray-drying, freeze-drying, coacervation, ionic gelation, crystallization, microencapsulation, electro-spinning, emulsification and extrusion-produced encapsulated bioactive compounds from agri-food wastes. The suitability of encapsulation techniques would be based on the entrapped method of bioactive compounds and their combination with the wall, encapsulating and carrier materials of different origins (like solution, polymers, an emulsion, dispersion, gelling etc.) and their applications [93]. Moreover, there is a dire need to study the bioavailability of bioactive compounds for food and agricultural applications, as well as production at pilot and commercial scales for industries. The encapsulation technique used for polymeric-based matrices is a good approach for bioactive compounds extracted from agri-food wastes and their applications in food and agriculture sector.

While the industrial application of encapsulation approaches still faces some limitations in the extraction, purification, identification, yield and technical costs of desired bioactive compounds from agri-food wastes, they are mostly low [94]. There is a dire need to develop new encapsulation technologies that are eco-friendly and cost-effective with low preparation requirements. In this context, continued scientific research will be required for improvement in advanced extraction and purification techniques for the development of suitable strategies for the sustainable use and utilizations of valuable bioactive compounds from agri-food wastes for further use in real life and their industrial applications [94,95,96].

## 7. Conclusions and Future Prospective

**Figure 10:**
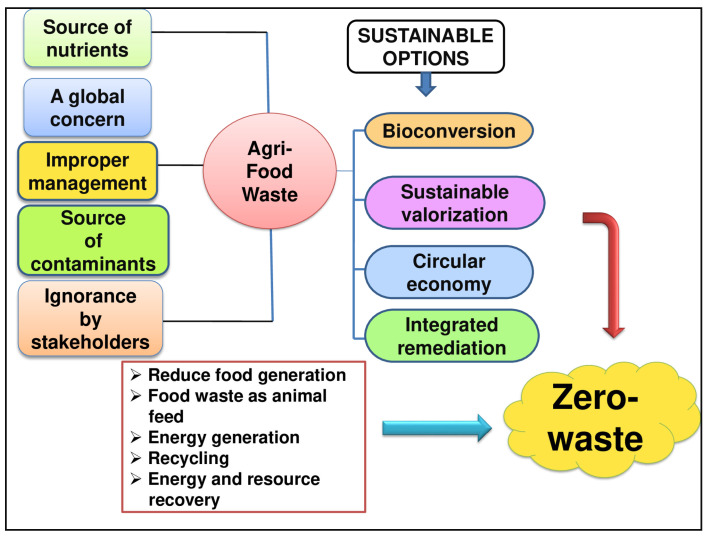
A schematic representation of current challenges, future perspectives and sustainable options for the valorisation of agri-food waste.

Agricultural waste constitutes a substantial reservoir of biomass that, while potentially causing economic and environmental challenges, is increasingly recognized as a valuable source of nutrition and functional raw materials with diverse applications [97,98]. This perspective presents a viable solution to both economic and environmental issues. This review primarily focuses on agricultural waste’s role in sustainable development, illustrating its potential for producing bioactive compounds that promote human health, improve everyday lifestyles and contribute to environmental pollution reduction through cost-effective and efficient methods. The processing of fruits and vegetables results in substantial waste that is replaced with bioactive compounds. Several bioactive compounds are derived from agri-food wastes and transformed into value-added products. The use of green, eco-innovative valorisation technologies is effective for minimizing waste, economically beneficial and health-promoting regarding the development of functional food ingredients and dietary supplements. Encapsulation is a novel and promising delivery system that could offer several benefits, but its limitations are still unclear for bioactive compounds. Because of the many benefits of encapsulation techniques, there are now various safety, regulatory and environmental concerns about how they may affect human health. Since no explicit international law has been implemented, their safety expectations are still unknown and need more investigation. Thus, future studies should emphasize the safety aspects and risk assessments of the use of micro- and nanoencapsulation of bioactive compounds in various applications; secondly, the improvement of existing encapsulation methods and their production on a commercial or industrial scale by optimizing different factors that affect the release of bioactive compounds for enhanced and more pronounced activity; and thirdly, the investigation of direct applications of bioactive compounds in the food industry and agriculture. New markets are being developed and current research is underway to reduce the high production costs and lack of food-grade materials used in the encapsulation of food. Moreover, effective policies and evaluations of their implementation over time can help mitigate economic and market resistance challenges. Recent years have seen continuous research efforts in improvement of innovative extraction and production techniques that would be acceptable for the nano-encapsulation strategies in a sustainable manner for maximum utilisation of agri-food wastes into valuable bioactive compounds and their application in the daily lives of humans and industries. In conclusion, improving and optimizing the isolation, extraction, processing and production of bioactive compounds from agri-food wastes and by-products via a sustainable approach is the need of the hour. Agri-food waste management and its valorisation through various sustainable approaches are concerned with the circular economy, socio-technical acceptance, green environment and recovery of valuable products into precious bioactive products. Figure 10 represents the current challenges and future perspectives with sustainable options for the valuation of agri-food waste globally.

Finally, this review highlighted the way for adopting sustainable extraction technologies for the conversion of agri-food waste through integrated valuation approaches towards valuable sources of bioactive compounds under waste into wealth or waste to value product (WtV) concepts on a global scale.

## Figures and Tables

**Table 1:** Application of non-conventional (green) advance extraction techniques for the recovery of bioactive compounds from substrates.

Agri-Food Waste (AFW)	Techniques	Extraction Conditions	Recovery	Reference
Rice grains Tomato skin Blueberry pomace Papaya peel Red beetroot juice	UAE	ET—45 °C Extraction time—25 min Solvent—methanol (80%) Ratio (solvent: solid)—5:1	Phenolic acids, anthocyanin, flavonol, carotenoids flavonoids	[23]
*C. asiatica* powder Potato peel *Picrasma* quassioides	MWAE UMAE (combination)	ET—30–35 °C Time—10 min Solvent—methanol Ratio (solvent:solid)—25:1 Isopropanol (solvent) (150 °C)	High yield of madicassoside, carotenoids, anthocyanin, phenols Highest amount of phenlic compounds	[24]
Sumac (*Rhus coriaria* L.) Tomato skin	SFE	ET: 40–42 °C Extraction Time: 45 min Solvent: Carbon dioxide + Ethanol	Extraction of high quercetin, carotenoids, phenolics	[25]
*Pinus pinaster* bark Blueberry pomace	PEFAE	Extraction Time: 30 min Extraction Solvent: 50% Ethanol Ratio (Solid-Solvent)—1:10, EC—12 mS/cm	Higher yields (up 30%) in comparison with conventional extraction, phenolic acids, anthocyanin	[26]
Pomegranate peel Onion skin	PLE	Extraction Temperature: 200 °C Extraction Time: 20 min Solvent: 77% Ethanol Pressure: 103 bar	Mainly ellagitannins extracted at high concentration, flavonoids	[27]
Chilean papaya (*Vasconcellea* *pubescens*)	HHPE	ET—room temp Time—10 min Pressure—500 MPa Solvent—Methanol (80%)	Four different phenolic compounds—caffeic, trans- ferulic and p-coumaric acids and rutin	[28]
Pomelo peel	EAE	ET—50 °C Time—60 min Enzyme—Pectinex Ultra SP-L (0, 1, 2, 3, or 4%, *v*/*w*)—0.9%	Total phenolic content higher as compared to others	[29]
Litchi peel	MAE + UAE	MAE—700C in 4 min of extraction with 40:1 solvent and material ratio	Pyrethroid	[30]
	UAE + PEFAE	UAE—3 min of extraction and chlorobenzene as a solvent, 30% aqueous ethanol, 62.66 mL/g ration of liquid: solid ratio, 123 mL/min flow velocity, 276 W ultrasonic probe, 47 °C ultrasonic temperature	Saponin	[31]
Lime peel	MAE + UAE	Microwave power 140 W with 55% ethanol and 45s ultrasound energy of 38% amplitude for 4 min	Phenolic compounds, antioxidants	[32]
Orange peel	EAE + PEFAE	High voltage of energy input of 222 kg/kg with enzymatic hydrolysis viscozyme of 12FBGU/g	Polyphenols and reducing sugars	[33]
Pomegranate seed	SFE + MAE + CE	Microwave radiation of 250 W with 6 min and then SFE and Soxhlet extraction	Punicic acid	[34]
Potato waste Yellow onion skin Red beetroot peel	CE	Maceration, hot water extraction, methanolic extraction	Phenolic, flavonoids, betalains	[34]
Grape peel	UAE + PEFAE	Ultrasonic energy with 50 °C with pulse of flow 290 L/h, diameter of chamber 25 mm, gap 26 mm and 25 kV voltage	Anthocyanin and flavonoid	[35]

ET—extraction temperature, UAE: ultrasound-assisted extraction; PLE—pressurized liquid extraction; SFE—supercritical fluid extraction; MWAE—microwave-assisted extraction; PEFAE—pulse electrical field-assisted extraction; UMAE—ultrasound microwave-assisted extraction, HHPE—high hydrostatic pressure extraction; EAE—enzyme-assisted extraction; CE—conventional extraction.

## Data Availability

No new data were created or analyzed in this study. Data sharing is not applicable to this article.
